# Macrophage Phagocytosis and Allergen Avoidance in Children With Asthma

**DOI:** 10.3389/fped.2018.00206

**Published:** 2018-08-02

**Authors:** Neeta Kulkarni, Ahmad Kantar, Silvia Costella, Vincenzo Ragazzo, Giorgio Piacentini, Attilio Boner, Christopher O'Callaghan

**Affiliations:** ^1^Department of Infection, Immunity and Inflammation, Institute for Lung Health, University of Leicester, Leicester, United Kingdom; ^2^Pediatric Cough and Asthma Center, Istituti Ospedalieri Bergamaschi, University and Research Hospitals, Bergamo, Italy; ^3^High Altitude Paediatric Asthma Centre in Misurina, Pio XII Institute, Belluno, Italy; ^4^Department of Pediatrics, Versilia Hospital, Lido di Camaiore, Italy; ^5^Pediatrics Section, Department of Surgery, Dentistry, Paediatrics, and Gynaecology, University of Verona, Verona, Italy; ^6^Respiratory, Critical Care and Anaesthesia, UCL Great Ormond Street Institute of Child Health, NIHR Great Ormond Street Hospital Biomedical Research Centre, Great Ormond Street Children's Hospital, London, United Kingdom

**Keywords:** macrophages, phagocytosis, asthma, children, allergen avoidance

## Abstract

**Background and Objective:** Airway macrophages perform the crucial functions of presenting antigens, clearing pathogens, and apoptotic cells. Macrophage phagocytosis is increased in adults with mild asthma and allergen exposure is known to activate macrophages. However, it is not clear whether the mechanism behind this is due to a primary defect or environmental factors such as allergen or lipopolysaccaride (LPS) exposure. Our aim was to assess the phagocytic function of airway macrophages in children with mild to moderate asthma after residence in a low allergen\LPS environment at high altitude.

**Methods:** Sputum induction was performed in children with asthma at baseline and after residence for a 3 weeks' period at a high-altitude asthma center that has very low ambient allergen levels. The markers of eosinophilic inflammation (including percentage of macrophage cytoplasm with red hue) and phagocytosis of fluorescein isothiocyanate-labeled, heat-killed *Staphylococcus aureus* by airway macrophages was analyzed. Internalized bacteria were quantified using confocal microscopy.

**Results:** The median bacterial count [mean (standard deviation)] per macrophage was significantly lower [39.55 (4.51) vs. 73.26 (39.42) (*p* = 0.006)] after residence at high altitude. No association was observed between markers of eosinophilic inflammation and bacterial phagocytosis.

**Conclusions:** The results suggest that the mechanism behind the enhanced phagocytosis of bacteria in childhood asthma may be secondary to allergen or possibly LPS exposure.

## Introduction

In recent years, knowledge of macrophage biology has vastly improved. The normally protective alveolar macrophages can turn into pathogenic cells in adverse lung environment demonstrating their plasticity (1). The transcriptome and epigenetic landscape of tissue macrophages have been demonstrated as being determined by the tissue-specific microenvironment. Macrophages are constantly sensing the milieu of the adjacent stroma to regulate the tissue homeostasis both during steady and inflammatory states. (2). Lavin at all have shown that local tissue environment can alter the macrophages to acquire the identity and function of tissue resident macrophages e.g., transfer of bone marrow derived alveolar macrophages or mature peritoneal macrophages into alveolar space converted to alveolar macrophage-like cells (3).

Irrespective of the origin of macrophages (fetal, adult or differentiated tissue) once in lung environment they can fully function as alveolar macrophages and are similar at genomic and epigenomic levels (4, 5). Various studies have revealed that the development and resolution of lung injury is accompanied by remarkable changes in the numbers and types of macrophages (6, 7).

The lung is exposed to the environmental toxins like, microorganisms, chemical agents (including environmental LPS), allergens, and antigens. The alveolar and airway macrophages perform a dual role of activation and suppression of inflammation (8).

Asthma research has long been focused on cells that appear to be the major contributors of airway inflammation, including eosinophils, neutrophils, and lymphocytes. However, few studies have endorsed the mechanisms of function and regulation of macrophages that may orchestrate the inflammatory process. Macrophages are front line defenders of innate immunity and also play a crucial role in organ development, tissue turnover, and regeneration (9). The macrophages are capable of sensing viral, microbial, parasitic antigens, immune complexes, and apoptotic or necrotic cells which helps in immune surveillance (9–11).

Airway macrophage phagocytosis in adults with mild asthma is increased compared to healthy individuals and is possibly related to higher levels of activation of macrophages (12). The enhanced phagocytic function could be related to increased levels of cysteinyl leukotrienes in allergic lung (13) or other inflammatory mechanisms. However, Alexis et al. (12) observed adults with mild asthma, and those with higher eosinophil counts had lower phagocytosis (12). This suggests that macrophage phagocytic function may be related to an interaction with allergen and eosinophilic inflammation. Thus far, there have been no studies on allergen exposure reduction to test this as a possible cause of enhanced phagocytosis. In addition, LPS is well known to activate macrophages resulting in increased chemotactic and phagocytic activity (14) and therefore reduction of LPS would reduce phagocytic activity.

Because of the low relative humidity at high altitudes, the environment is free of house dust mites and molds (15). A number of studies in children (16–20) and adults with asthma have revealed a beneficial effect of high altitude residence on symptoms, lung function, and eosinophilic inflammation (21). However, no study has explored the effect of high altitude on macrophage phagocytosis, which results in a marked reduction in allergen and also possibly LPS exposure.

In adults with high levels of airway eosinophilic inflammation (defined as a sputum eosinophil count greater than 5%), macrophage phagocytosis has been shown to be lower, indicating that eosinophilic inflammation may affect phagocytosis (12). The toxic effect of eosinophil proteins on the epithelium and other cells (22, 23) is well known. These proteins may impair macrophage function.

We have previously shown, however, that sputum eosinophil count alone may be insufficient to identify ongoing eosinophilia (24). In children with asthma glucocorticoid treatment results in reduction of sputum eosinophil count, this could also be due to drop in recruitment of eosinophils because of natural resolution of disease process. Corticosteroid therapy increases eosinophil apoptosis and uptake by macrophages, thereby increasing the eosinophil protein content within the macrophages. To differentiate between these processes and to identify ongoing eosinophilia, we recently developed a novel marker of eosinophilic inflammation (eosinophil protein content in airway macrophages) (24).

In this study we hypothesized that airway macrophage phagocytosis would be lower after reduction of allergen exposure in children with mild to moderate asthma. To test our hypothesis, we compared airway macrophage phagocytosis before and after allergen avoidance during residence at the High Altitude Paediatric Asthma Centre in Misurina (1,756 m), which offers residential treatment for children with asthma. In addition, we assessed the relationship between airway macrophage phagocytosis and markers of eosinophilic inflammation (including eosinophilic proteins in airway macrophages) before their high-altitude residence. According to our understanding, this is the first study that explores the effect of a high altitude, with allergen and possibly LPS avoidance, on the phagocytic function of sputum macrophages in pediatric or adult asthma.

## Materials and methods

### Study patients

The study enrolled children aged 7–17 years (*n* = 62) admitted to Istituto Pio XII, Misurina, Belluno (High Altitude Paediatric Asthma Centre) from various cities in Italy between June and September 2010. Children with a diagnosis of asthma and residing in the center for 3 weeks or more were included. The diagnosis of asthma was supported by clinical symptoms and reversibility testing (> 12% increase in FEV_1_ after short acting bronchodilator treatment) (25). The exclusion criteria were respiratory infection in the preceding 6 weeks, congenital heart disease and chronic suppurative lung disease, or associated respiratory conditions such as cystic fibrosis and primary ciliary dyskinesia.

### Study design

On the day of arrival (T0) the demographic data, exposure to tobacco smoke, and medications including the doses of inhaled steroids were recorded. All children underwent clinical examination; spirometry, FeNO, and skin prick tests were performed. Spirometry was carried out as per the American Thoracic Society Guidelines (26). Sputum induction and blood eosinophil blood counts were performed within 2 days of arrival. At the end of the stay (T1) the symptoms during the stay and the treatment received were recorded and sputum induction was performed. The inhaled corticosteroid dose was converted to an estimated equipotent daily dose, in accordance with the guidelines of the Global Initiative for Asthma (GINA) (25) to compare the groups. Ethical approval was obtained by the Ethics Committee for Clinical Research of the Local Heath Authority in Belluno, and the parents provided written informed consent for the study. Our investigation was restricted to children with asthma because no healthy children resided at a high altitude for the time required by the study.

## Methods

### Reagents and chemicals

All reagents, culture media, and latex beads were purchased from Sigma–Aldrich (Milan, Italy) unless otherwise specified.

## Exhaled nitric oxide measurement

FeNO was measured using a standard technique complying with the recommendations of the European Respiratory Society/American Thoracic Society (27), using a chemiluminescence analyzer (Logan LR 2149; Logan Research Ltd., Rochester, Kent, UK), and expressed as parts per billion (ppb).

## Sputum induction and processing

Sputum was induced and processed as described previously (28, 29). Air-dried cytospins were stained with Diff-Quik. The total cell count, cell viability, and level of squamous cell contamination were assessed. The eosinophil differential count was obtained by counting 400 non-squamous cells and expressed as a percentage. The children were divided into 2 groups depending on the differential count on arrival (T0): eosinophilic (≥ 3%) and non-eosinophilic (< 3%).

### Macrophage eosinophil protein content

The image analysis method used was as previously described (24). Please refer to the online supporting information for details.

### Macrophage culture and phagocytosis assays

The macrophage culture and phagocytosis are described in the online supporting information.

## Confocal microscopy and image analysis of *staphylococcus aureus* phagocytosis

After adherence, macrophages were incubated for 2 h with fluorescein isothiocyanate conjugated, heat-killed *Staphylococcus aureus* (Invitrogen Milan Italy) resuspended in RPMI 1640 supplemented with 5% FBS (10:1 ratio of staph aureus/Airway Macrophage). Please refer to online supporting information for details of further processing of sample, confocal microscopy, and image analysis to quantify internalized bacteria. The median bacterial count/airway macrophage and median maximum intensity\airway macrophage was calculated for each subject.

### Latex bead phagocytosis: phagocytic index

After adherence, macrophages were incubated with 2-μm latex beads (airway macrophage: bead = 1:10) resuspended in RPMI 1640 (supplemented with 5% FBS) for 2 h. Please refer to the online supporting information for details on the counting of internalized bacteria. The phagocytic index (beads/100 airway macrophages) and number of phagocytic macrophages were calculated.

### Statistics

Statistical analysis was performed using GraphPad Prism 6 (GraphPad, San Diego, CA, USA). Patient characteristics are presented as mean (standard error), median (range or interquartile range) or percentages. The between-group comparisons for non-parametric data were made using the Mann–Whitney test for unpaired data and the Wilcoxon matched-pair test for paired data as appropriate and proportions were assessed using Fisher's exact/chi-square test. Differences were considered significant when *p* < 0.05. Linear regression and Spearman's co-relations were used to explore the association between the macrophage eosinophil protein content and phagocytosis.

## Results

### Patient characteristics

The clinical characteristics of 54 children who produced adequate sputum for cell counts on arrival (T0) are as shown in Table 1. All children were in GINA Groups 1–3 (with 77.7% of children in GINA Groups 1 and 2) depending on the treatment in the previous 3 months prior to the start of residency. Thirty children had sputum eosinophil counts over 3% and were therefore assigned to the eosinophilic group, with 24 in the non-eosinophilic group. The median (range) sputum eosinophil content in the eosinophilic group was 12% (3–52%). Boys were predominant in the eosinophilic group. No difference was observed in FEV_1_ between the groups. However, the predicted FEF_25−75_% was lower and blood eosinophil and FeNo content were higher in the eosinophilic phenotype (Table 1).

**Table 1:** Baseline characteristics of patients.

	**Eosinophilic group** **(*n* = 30)**	**Non-eosinophilic group** **(*n* = 24)**	***p*-value**
Mean age (yr) (SD)	13.51 (± 3.1)	13.02 (± 2.9)	0.55
Sex (male), No. (%)	25 (83.3)	13 (54.1)	**0.034**
BMI	18.99 (14.4–31.9)	19.99 (12.8–41.1)	0.7
Smoking in house (yes) No. (%)	12 (40)	7 (29.2)	0.56
Food allergy (yes) No. (%)	14 (46.6)	9 (37.5)	0.58
GINA Classification No. (%)			0.30
1	6 (20)	9 (37.5)	
2	17 (56.7)	10 (41.7)	
3	7 (23.3)	4 (16.7)	
5	0	1 (4.1)	
FEV_1_ % predicted mean (SE)	106.8 (2.97)	113.7 (3.7)	0.13
FVC% predicted mean (SE)	104.5 (2.22)	107.2 (2.8)	0.45
FEF_25−75_% predicted Mean (SE)	98.27 (5.03)	115.9 (6.5)	**0.034**
Blood eosinophils (%)	7.8 (3.9–12.1)	5.05 (1.9–13.1)	**0.014**
Median FeNo (ppb) (range)	23.75 (6.3–79.3)	12.8 (12.7–41.1)	**0.001**
**MEDIAN INDUCED SPUTUM (RANGE)**			
Total cell count × 10^6^ \g sputum	2.3 (0.39–9.5)	1.7 (0.05–7.2)	0.48
Cell viability (%)	86.5 (59.7–96.4)	88.9 (57.9–100)	0.27
Macrophages (%)	62.5 (1–88)	72.5 (14–97)	0.17
Neutrophils (%)	22 (8–72)	26.5 (3–86)	0.46

*(Eosinophilic group ≥ 3% sputum eosinophil differential count). Bold P-values indicates p < 0.05*.

**Figure 1:**
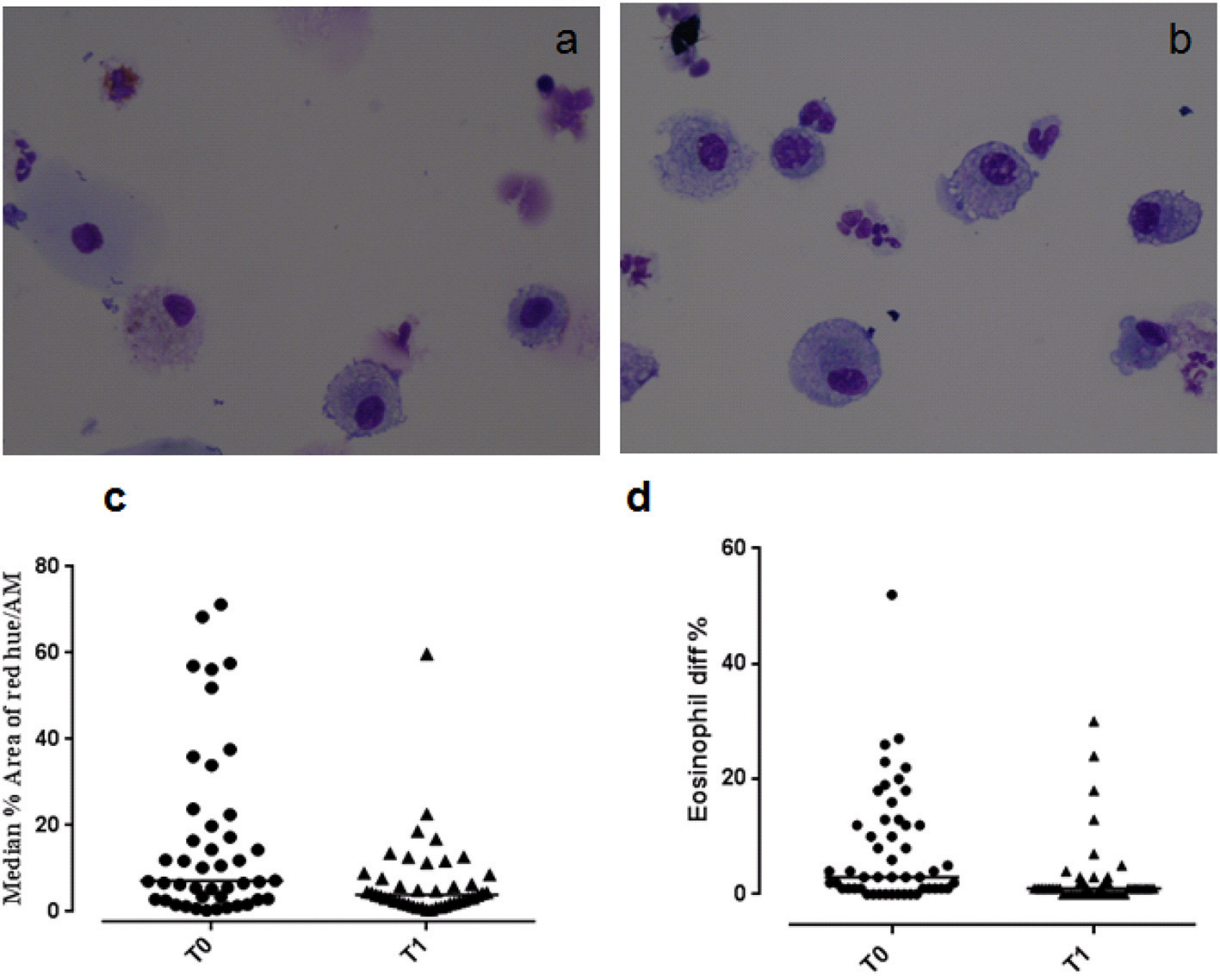
Eosinophilic staining of macrophages at **(a)** T0 and **(b)** T1. Graphs showing **(c)** the median % red hue/airway macrophage ratio, and **(d)** sputum eosinophil differential counts at T0 and T1.

Fifty children produced adequate sputum samples on both occasions (T0 and T1) for differential counts. No statistical difference was observed in lung function measurements before or after their high-altitude stay in a very low ambient allergen environment. However, after exposure to a high altitude (T1), they had a significantly lower FeNO (*p* < 0.001) and eosinophil counts (*p* = 0.001) (Table 2, Figure 1d). No difference was observed in percentage cell viability (Table 1) (all cell types) in the eosinophilic and non-eosinophilic groups or in the T0 or T1 samples. The patients who produced an adequate sample for phagocytosis assay did not differ from those who did in age, lung function parameters, blood eosinophil counts, or sputum cell counts. The equipotent inhaled corticosteroid dose was reduced (*p* = 0.01) (Table 2) during their stay at the center because the children were exhibiting fewer symptoms.

**Table 2:** Lung function, FeNo, sputum differential counts, inhaled corticosteroids, and macrophage red hue percentage results on arrival (T0), and after the allergen reduction intervention (3-week residence at a high-altitude asthma center) (T1).

	**T0** **(*n* = 50)**	**T1** **(*n* = 50)**	***p*-value**
FEV_1_% predicted mean (SE)	109.5 (2.4)	116.0 (2.04)	0.67
FVC% predicted mean (SE)	105.8 (1.81)	105.6 (1.89)	0.91
FEV/FVC ratio % predicted mean (SE)	103.1 (1.29)	101.8 (1.3)	0.56
FEF_25−75_% predicted mean (SE)	105.3 (4.38)	102.9 (4.01)	0.72
Median FeNo (ppb) (range)	18.85 (2.6–79.3)	11.5 (2.1–52.2)	**0.0004**
ICS treatment^*^			**0.01**
No ICS treatment No. (%)	13 (26)	20 (40)	
Low daily dose No. (%)	21 (42)	26 (52)	
Medium daily dose No. (%)	16 (32)	4 (8)	
**MEDIAN INDUCED SPUTUM (RANGE)**			
Total cell count × 10^6^\g sputum	2.2 (05–9.5)	2.25 (0.26–11.2)	0.24
Macrophages (%)	65 (1–97)	63 (6–87)	0.15
Neutrophils (%)	24.5 (3–86)	32.5 (11–90)	**0.006**
Eosinophils (%)	3 (0-52)	1 (0–30)	**0.001**
Lymphocytes (%)	0 (0–2)	0 (0–8)	0.7
Median % area red hue/airway macrophage (range) (*n* = 43 pairs)	7.04 (0.72–71.23)	3.82 (0.41–59.82)	**0.005**

* *GINA classification of equipotent daily dose of inhaled glucocorticosteroids for children older than 5 years (25). Bold P-values indicates p < 0.05*.

### Macrophage eosinophilic proteins

Example images of red hue in macrophages before and after residence in Misurina, as an index of eosinophil uptake, are shown in Figures 1a,b. No significant difference was observed (*p* = 0.39) between median airway macrophage red hue percentage in eosinophilic (*n* = 30) [median (range)] [5.9 (0.6–57.6)] and non-eosinophilic asthma (*n* = 24) [8.7 (0.34–71.2)]. After time spent at a high altitude (T1), the children (*n* = 43 pairs) had a significantly lower airway macrophage red hue percentage [*p* = 0.005] than before T0 (Table 2, Figure 1c).

### Macrophage phagocytosis

**Figure 2:**
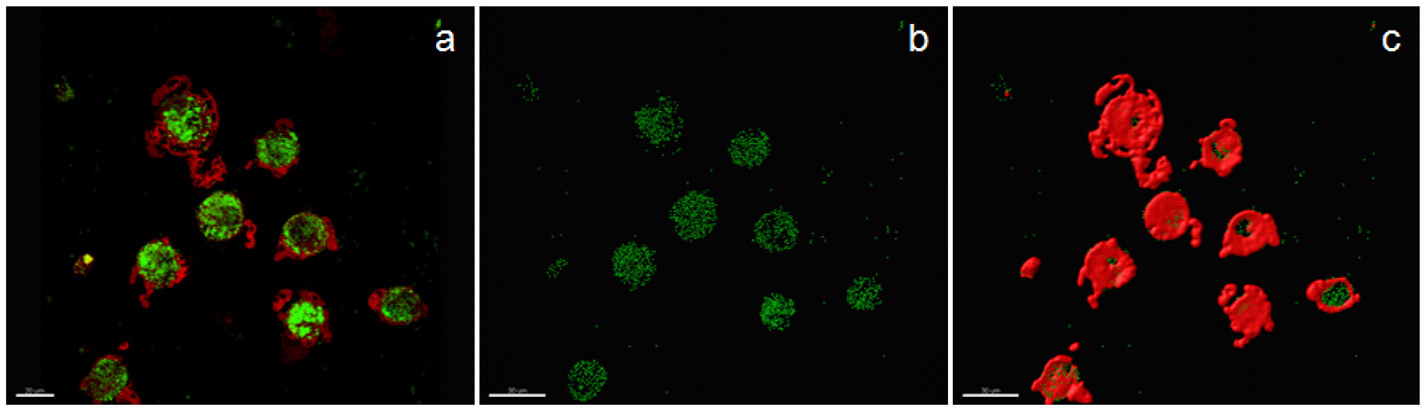
Confocal image of sputum macrophages cultured with heat-killed fluorescein isothiocyanate-labeled *Staphylococci aureus* (airway macrophage:bacteria = 1:10) showing internalized bacteria. **(a)** Confocal image of bacteria (green) and cytoplasm (red), **(b)** bacteria alone, and **(c)** the surface view of macrophages showing that a majority of the bacteria are internalized.

**Figure 3:**
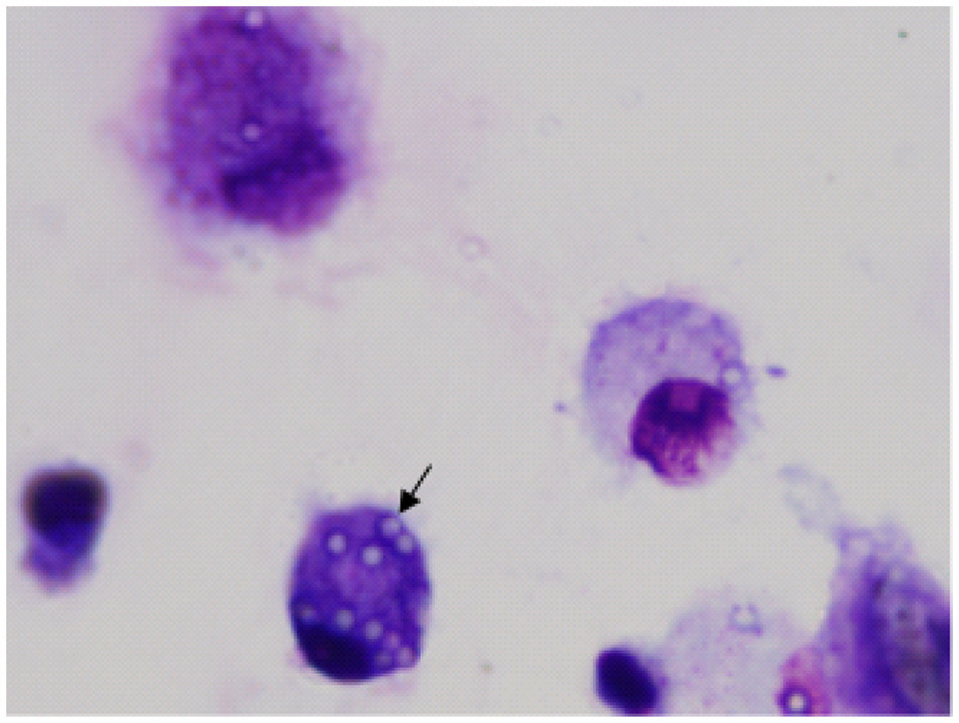
Image of airway macrophages cultured with latex beads (airway macrophage: bead = 1:10) for 2 h. External beads were digested with xylene. Internalized beads are preserved (arrow).

Examples of confocal images showing internalized bacteria and light microscopy images showing latex beads are presented in Figures 2, 3, respectively. No significant difference (*p* = 0.3) was observed between the median bacterial counts in eosinophilic (*n* = 16) and non-eosinophilic asthma (*n* = 8) at T0. After exposure to a high altitude (T1), the children (*n* = 19 pairs) exhibited significantly lower median bacterial counts [Mean (standard deviation)] [*p* = 0.006, 39.55 (4.51) vs. 73.26 (39.42)] than at T0 (Table 3).

**Table 3:** Phagocytosis assay on arrival (T0) and after the allergen reduction intervention (T1) (3-week residence at a high-altitude asthma center).

**Bacterial phagocytosis** **(*n* = 19 pairs)**	**T0**	**T1**	***p***
Mean bacterial count mea (SD)	73.26 (39.42)	39.55 (4.51)	**0.006**
Mean max intensity (SD)	0.044 (0.010)	0.038 (0.01)	0.08
**LATEX BEAD PHAGOCYTOSIS (*****n*** = **10 pairs)**			
Median beads/100 cells (range)	201.0 (71.56–1268)	44.52 (9.8–246.8)	**0.003**
Mean % phagocytic AM (SD)	57.70 (16.24)	27.86 (16.36)	**0.001**

*Bold P-values indicates p < 0.05*.

### Eosinophilic inflammation and phagocytosis

No significant correlation or association was observed between age, FeNo, pre- and post-bronchodilator lung function (FEV_1_% predicted, FVC% predicted, FEF_25−75_% predicted), blood counts (total white count, eosinophil count), or sputum parameters (viability, eosinophil and neutrophil counts) and bacterial uptake by macrophages (median bacterial count) and median maximum intensity (bacterial fluorescence). Latex bead phagocytosis (number of phagocytic macrophages (%) and phagocytic index) were significantly positively associated with FeNo (*p* = 0.028) and blood eosinophils (*p* = 0.028), but were not associated with any other parameter. There was a positive correlation between number of phagocytic macrophages (%) and blood eosinophils (*r* = 0.508, *p* = 0.046). The median airway macrophage red hue area percentage was not associated with bacterial or latex phagocytosis parameters. Please refer to online Supplement Figures 1–4.

## Discussion

Alveolar macrophages line the luminal surface and are primary defense mechanism to protect the lungs from invading pathogens and pollutants. They initiate and evolve the immune response in the lung. Overall, the number of macrophages in patients with asthma is similar to that in healthy individuals (30).

In asthma, two major functionally distinct subsets of macrophages have been intensively investigated: the M1 and M2 macrophages. Macrophages have been grouped into M1 and M2 types depending on their function in subjects with asthma. M1 macrophages are differentiated by bacteria-derived mediators such as interferon-c and lipopolysaccaride (LPS) and release various inflammatory cytokines and chemokines, whereas M2 macrophages have increased phagocytic activity but are poor at clearing intracellular pathogens. Moreover, experimental data suggests both M1 and M2 subsets are involved in asthma (31). In addition there are regulatory macrophages performing different physiological functions and develop in response to different stimuli (32). These are vital in regulating immune responses and reduce inflammation (33). Macrophages are critical for both innate and acquired immunity and play a pivotal role in lung defense. Lung macrophages recognize a wide variety of pathogenic antigens, immune complexes, and apoptotic or necrotic cells (11). Increased infection in asthma has led to increased investigation of the clearance of bacterial, cellular, and inhaled particles by macrophages in patients with asthma.

Alexis et al. have demonstrated that phagocytosis of opsonised particles in subjects with mild asthma with airway eosinophilia was lower as compared to those without eosinophilia. Overall phagocytosis was comparable between controls and those with mild intermittent asthma (12). In a similar subjects, phagocytic activity in airway macrophages decreased by about 50% 6 h after endotoxin (LPS) inhalation (34).

Fitzpatrick et al. sampled bronchoalveolar lavage macrophages from children with severe asthma and demonstrated impaired phagocytosis of *S. Aureus*. Interestingly this remained unchanged after LPS stimulation. Moreover, they have shown that children with severe asthma also had increased alveolar macrophage apoptosis (35). These later findings were attributed to imbalance in glutathione homeostasis (36). Similar findings of reduced phagocytosis of *H influenzae* and *S aureus* were seen in bronchoalveolar lavage and monocyte-derived macrophages from adults with severe asthma (37). Not only bacterial uptake but apoptotic cell clearance by lavage macrophages is reduced in severe asthma as compared to healthy controls or mild-moderate asthma patients (38).

Studies have demonstrated that severe asthma is associated with inflammatory mediators such as prostaglandin E2 and D2, which can suppress the phagocytic activity of alveolar macrophages (39–41). Recently, Brugha et al. investigated the phagocytosis of inhalable carbonaceous particle matter by airway macrophages obtained from the induced sputum of asthmatic children. Their results demonstrated a 51% lower carbon content in the macrophages of children with moderate to severe asthma compared with mild asthma and healthy children (42). Moreover, the study demonstrated an inverse association between alveolar macrophage carbon and urinary metabolites of prostaglandin E2 and prostaglandin D2.

The effect of a high altitude on improvement of asthma symptoms has been attributed to the very low levels of ambient allergens. In this study, we described for the first time the effect of residence at a high altitude with markedly reduced allergens and possibly pollutants (e.g., LPS) on the phagocytic function of sputum macrophages and markers of eosinophilic inflammation in children with mild to moderate asthma. According to our understanding, this is the original study exploring the effect of reduced allergen\LPS exposure on the phagocytic function of sputum macrophages in pediatric or adult asthma. The phagocytosis of heat-killed bacteria in this group of children decreased after residence at a high altitude and was similar in eosinophilic and non-eosinophilic phenotypes. We also found that bacterial phagocytosis was not associated with parameters of eosinophilic inflammation, including the median airway macrophage red hue percentage.

Our study strongly suggests that allergen and possibly LPS exposure is a major contributor to enhanced macrophage phagocytosis in asthma. The induced sputum macrophage capacity to phagocytose particles has been demonstrated to be higher in subjects with mild asthma (12, 43) than in healthy controls. One possible explanation is that activation of macrophages is enhanced in asthma. Silva et al showed that cysteinyl leukotrienes enhanced Fcγ-mediated phagocytosis in sensitized rats (13). **(author?)** (28) showed that children with asthma, after residence in a very low ambient allergen environment at a high altitude had lower exhaled breath cysteinyl leukotrienes and leukotriene B4 (28). This might partially explain our finding of reduced phagocytosis in children with mild asthma staying in similar conditions. Along with reduction in allergens there is also possibility of lower level of ambient pollution including LPS. LPS is known to stimulate macrophages and therefore reduction would lower phagocytic capacity.

Fitzpatrick et al. sampled bronchoalveolar lavage macrophages from children with severe asthma and demonstrated impaired phagocytosis (35) but did not include children with mild asthma. We were unable to compare our results of phagocytosis assays with other studies assessing sputum macrophage phagocytosis because this is the first such study on children with asthma. In addition, no studies have included reduction of allergens as an intervention to assess phagocytosis of sputum macrophages. No difference was observed in the viability of sputum cells on arrival or after residence at a high altitude that could have affected the macrophage phagocytic function.

We recently described a novel marker (macrophage eosinophil protein content measured as red hue percentage) that identifies ongoing sputum eosinophilia in patients with asthma and a normal sputum eosinophil count (24). On admission, children without sputum eosinophilia had similar airway macrophage red hue percentages as those with sputum eosinophilia. This suggests the presence of ongoing eosinophilia in the non-eosinophilic group. Because airway macrophages acquire eosinophilic proteins by phagocytizing apoptotic eosinophils, a lower airway macrophage red hue suggests that macrophages have ingested fewer eosinophils. Therefore, the combination of low airway macrophage red hue and low sputum eosinophil counts suggests a reduction of eosinophil recruitment to the airways. The reduction of sputum eosinophilia after residence at a high altitude has previously been demonstrated (20, 28). In children with asthma glucocorticoid treatment results in reduction of sputum eosinophil count, this could also be due to drop in recruitment of eosinophils because of natural resolution of disease process. Corticosteroid therapy increases eosinophil apoptosis and uptake by macrophages, thus increasing the eosinophil protein content within macrophages. It could be argued that when admitted to the center that supervises administration of treatment, the improved adherence to therapy could have contributed to the reduction in eosinophilic inflammation. However, this is unlikely because the airway macrophage red hue percentage dropped after residence at a high altitude, suggesting reduction of eosinophil ingestion by sputum macrophages and reduced recruitment. Therefore, using the macrophage red hue measurement has the added value of helping to differentiate between a reduced sputum eosinophil count related to treatment and that related to reduced recruitment of eosinophils.

Although evidence (12) indicates that increased eosinophilic inflammation is inversely associated with phagocytosis, we did not find an association between bacterial phagocytosis and markers of eosinophilic inflammation (including the eosinophilic proteins in airway macrophages). However, this relationship must be explored further in children with severe asthma who have persistent inflammation.

Our study had some limitations. We were unable to recruit healthy children because they did not reside for 4 weeks at the high-altitude asthma center. Because of the small amount of sputum samples obtained from the children, insufficient numbers of macrophages were obtained to analyze the macrophages for cell surface markers of activation. It is possible that *in vivo* phagocytosis may be affected by other factors that we did not measure. However, our study results indicating higher phagocytosis in patients with mild asthma are like those of inhaled particles in adults in *in vivo* experiments (43).

In conclusion, induced sputum was successfully used to study macrophage phagocytosis in children with asthma. The sputum macrophages in children with mild to moderate asthma after allergen and possibly lipopolysaccaride avoidance at a high altitude are less phagocytic. Because activation of macrophages by allergens in mild asthma enhances phagocytosis, removal of this stimulus appears to reduce phagocytosis. No association was observed between phagocytosis and eosinophilic inflammation markers. Further research is essential to dissect the mechanism of various types of alveolar macrophage phagocytosis in asthma.

## Author contributions

All authors listed have made a substantial, direct and intellectual contribution to the work, and approved it for publication.

### Conflict of interest statement

The authors declare that the research was conducted in the absence of any commercial or financial relationships that could be construed as a potential conflict of interest.
