# Supplementary material for: Macrophage Phagocytosis and Allergen Avoidance in Children With Asthma
Source: Front Pediatr. 2018 Aug 2;6:206. doi: 10.3389/fped.2018.00206 (PMC6082964; doi:10.3389/fped.2018.00206)
Supplement: Supplementary file 1 [file Table_1.DOCX]

# Online supplement

# MACROPHAGE PHAGOCYTOSIS AND ALLERGEN AVOIDANCE IN CHILDREN WITH ASTHMA

**Short title: Macrophage phagocytosis and allergen avoidance**

**Macrophage eosinophil protein content**

The method of image analysis was used as we previously described^1^. In brief, from the cytospins 100 macrophages were imaged using x40 objective at ultrahigh resolution (4080x 3072) (Olympus DP70 digital camera, AnalySIS, Soft Imaging System GmbH, Germany). Image J software (ImageJ 1.40g/java 1.6.0_05, NIH Image) was used to analyse the percentage area of cytoplasm with red hue by thresholding. Median percentage area of cytoplasm with red hue (primary measure) in 100 macrophages per subject was used as a measure of eosinophil protein content.

**Macrophage culture and phagocytosis assays**

The macrophage count was performed using Kimura stain. The cells were resuspended in RPMI 1640 supplemented with 5% Foetal Bovine Serum (FBS) (Invitrogen Milan Italy). 3x 10^5^ macrophages were added to each well of 8 chamber permanox slide (Nunc VWR Milan Italy) and allowed to adhere for 2 hours at 37°C in 5% CO_2_.

**Staphylococcus aureus phagocytosis using confocal microscopy and image analysis**

After adherence macrophages were incubated for 2 hours with Fluorescein isothiocyanate –conjugated Heat killed Staphylococcus aureus (Invitrogen Milan Italy) resuspended in RPMI 1640 supplemented with 5% FBS (10:1 ratio of staph aureus/AMs). Extracellular fluorescence was quenched by using 4% Trypan blue for 1 min. Cells were fixed with 4% Paraformaldehyde. Nuclei were stained with DAPI and cell cytoplasm by incubation with Evans blue dye (0.1% w/v). Bacterial phagocytosis was quantified using confocal microscope (Nikon Eclipse T1 Surrey UK) containing Argon/krypton laser. The EZC1 software (Version 3.9) was used to capture 50 AM from each slide. For each channel, the maximum intensity image in the stack was converted to single collapsed image using Image J (ImageJ 1.40g/java 1.6.0_05, NIH Image). The cells were identified and bacteria counted using CellProfiler software (Version 10415). An in house macro/ pipeline were built to use in both the software’s. The median bacterial count/AM and median maximum intensity\AM was calculated for each subject.

**Latex bead phagocytosis: Phagocytic index**

After adherence macrophages were incubated with 2 µm latex beads (AM:bead = 1:10) resuspended in RPMI 1640 supplemented with 5% FBS were added and incubated for 2 hours. Phagocytosis was suspended by adding cold PBS. Macrophages were detached by scrapping and cytospins prepared. The air-dried slides were stained with Diff-Quik and DPX was used as mountant before using coverslip. The xylene in the mounting media dissolved the non- phagocytosed/ adhered latex beads. The number latex beads/AM were counted using light microscopy under oil immersion. The phagocytic index (beads/100 AM) and number of phagocytic AM were calculated.

Reference

[1] Kulkarni NS, Hollins F, Sutcliffe A, Saunders R, Shah S, Siddiqui S, Gupta S, Haldar P, Green R, Pavord I et al. Eosinophil protein in airway macrophages: a novel biomarker of eosinophilic inflammation in patients with asthma. J Allergy Clin Immunol 2010;126:61-69 e63.

**Supplement Figure legends**

The graphs showing relationship of phagocytosis and eosinophilic markers [**l**inear regression (p) and Spearman Correlation Coefficient (ρ)]

**Supplement Figure 1**: Graphs showing median bacterial count\Airway macrophage (AM) and A) FeNo B) blood eosinophils (%) C) Sputum Eosinophil differential count (%) D) Median % Area of red hue/AM

**Supplement Figure 2**. Graphs showing median maximum intensity\AM and A) FeNo B) blood eosinophils (%) C) Sputum Eosinophil differential count (%) D) Median % Area of red hue/AM

**Supplement Figure 3**. Graphs showing Latex bead phagocytic Index (PI) and A) FeNo B) blood eosinophils (%) C) Sputum Eosinophil differential count (%). D) Median % Area of red hue/AM.

**Supplement Figure 4**. Graphs showing percentage of AM with latex bead phagocytosis and A) FeNo B) blood eosinophils (%) C) Sputum Eosinophil differential count (%) D) Median % Area of red hue/AM

**Supplement Figure 1**.

1. **B)**

**C)** **D)**

**Supplement Figure 2**

1. **B)**

**C) D)**

**Supplement Figure 3**

**A) B)**

**C) D)**

**Supplement Figure 4**

**A) B)**

**C) D)**
